# Trace element accumulation in lotic dragonfly nymphs: Genus matters

**DOI:** 10.1371/journal.pone.0172016

**Published:** 2017-02-16

**Authors:** Dean E. Fletcher, Angela H. Lindell, Garrett K. Stillings, Susan A. Blas, J. Vaun McArthur

**Affiliations:** 1 Savannah River Ecology Laboratory, University of Georgia, Aiken, South Carolina, United States of America; 2 Area Completion Projects, Savannah River Nuclear Solutions, Aiken, United States of America; Seoul National University, REPUBLIC OF KOREA

## Abstract

Constituents of coal combustion waste (CCW) expose aquatic organisms to complex mixtures of potentially toxic metals and metalloids. Multi-element trace element analyses were used to distinguish patterns of accumulation among 8 genera of dragonfly nymphs collected from two sites on a CCW contaminated coastal plain stream. Dragonfly nymphs are exceptional for comparing trace element accumulation in syntopic macroinvertebrates that are all predators within the same order (Odonata) and suborder (Anisoptera), but differ vastly in habitat use and body form. Sixteen trace element (Be, V, Cr, Ni, Cu, Zn, As, Se, Sr, Cd, Sb, Cs, Ba, Hg, Tl, and Pb) were analyzed and trophic position and basal carbon sources assessed with stable isotope analyses (C and N). Trophic positions varied within relatively narrow ranges. Size did not appear to influence trophic position. Trophic position rarely influenced trace element accumulation within genera and did not consistently correlate with accumulation among genera. Patterns between *δ*^13^C and trace element accumulation were generally driven by differences between sites. An increase in trace element accumulation was associated with a divergence of carbon sources between sites in two genera. Higher trace element concentrations tended to accumulate in nymphs from the upstream site, closer to contaminant sources. Influences of factors such as body form and habitat use appeared more influential on trace element accumulation than phylogeny for several elements (Ni, Ba, Sr, V, Be, Cd, and Cr) as higher concentrations accumulated in sprawler and the climber-sprawler genera, irrespective of family. In contrast, As and Se accumulated variably higher in burrowers, but accumulation in sprawlers differed between sites. Greater variation between genera than within genera suggests genus as an acceptable unit of comparison in dragonfly nymphs. Overall, taxonomic differences in trace element accumulation can be substantial, often exceeding variation between sites. Our results underscore the element and taxa specific nature of trace element accumulation, but we provide evidence of accumulation of some trace elements differing among dragonflies that differ in body form and utilize different sub-habitats within a stream reach.

## Introduction

When exposed to multiple element contaminations, increased trace element accumulation can be a near community wide response [1]. However trace element accumulation in aquatic macroinvertebrates can exhibit both element and species specific patterns [1, 2, 3, 4]. No single macroinvertebrate taxa will likely be consistently high or low in all essential or non-essential elements, but multi-element trace element concentrations can distinguish patterns in accumulation among taxa and sites [5, 6, 7]. Constituents of coal combustion wastes (CCW) exposes aquatic organisms to a such broad variety of potentially toxic metals and metalloids such as As, B, Cd, Cr, Cu, Hg, Ni, Pb, Sb, Se, Sr, V, and Zn [8, 9, 10]. Release of coal combustion wastes (CCW) from coal-fired electricity generating plants into aquatic systems represents a global environmental problem [9, 11, 12, 13, 14]. In the United States alone, millions of tons of CCW have accumulated for decades at onsite surface ponds that are broadly distributed across the country [8, 15]. Disposal of CCW into aquatic surface impoundments has led to contamination of surface waters and consequent toxicological effects on wildlife inhabiting these systems (reviewed in [10, 16].

Coal-fired power plants operated on the Savannah River Site, SC, USA for over 50 years during which substantial releases of CCW in aquatic habitats occurred [9, 17]. Such releases in our study system, Beaver Dam Creek (BDC), have exposed aquatic organisms to suites of elements such as Al, Ba, Fe, Hg, Mn, Sr, V, Cu, Cr, Cd, Sb, As, Ni and Zn [9, 17, 18, 19, 20, 21, 22]. Exposure to such a broad suite of elements provides opportunity to investigate element and taxa specific patterns in accumulation. Aquatic invertebrate accumulation in BDC has been previously reported for the heptageniid mayfly, *Maccaffertium modestum* [23] and Asiatic clam, *Corbicula fluminea* [23, 24]. Additionally, accumulation in *C*. *fluminea*, the snail *Helisoma* and two dragonfly genera (*Tramea* and *Erythemis*) was reported in wetlands in the headwaters of BDC [21]. Accumulation of CCW elements in a variety of aquatic invertebrates was reported in floodplain wetlands connected to BDC [25, 26, 27].

Dragonflies are excellent models for diverse ecological, evolutionary, and behavioral studies [28]. The utility of dragonflies in assessments of bioavailable contaminants [6, 29, 30, 31, 32, 33] and stream assessments [34] is becoming increasingly appreciated. Their predaceous behavior and relatively long nymph stage renders them potentially excellent integrators of biologically available contaminants across time. Total contaminant body burden has been shown to vary in element and taxon specific manners [33, 35]. This follows broad patterns of high variability of accumulation among aquatic invertebrates revealed by evaluating varying uptake rates from solution and diet, amplified by varying efflux rates [1, 2, 3, 4, 36].

Phylogenetically based patterns in trace element efflux or accumulation have been noted in aquatic macroinvertebrates, but even closely related taxa can vary in trace element accumulation [1, 4, 36]. Dragonfly nymphs provide an exceptional opportunity to compare accumulation of contaminants in macroinvertebrates that are all predators within the same order (Odonata) and suborder (Anisoptera), but differ vastly in their body form, hunting behaviors, and habitat use. Body form ranges from long and slender to broad, palmate abdomens and from visual predators to tactile sensory detection of prey [37]. Legs can be short and compact to long and spider-like. Differentiation in body form may influence levels of contaminant adsorption or absorption. Ecological habits range from burrowing in the bottom sediments, through sprawling across the surface sediments, to clinging to suspended wood debris, vegetation, or root masses. Niche breadth can range from highly specific to more broadly distributed generalists [38]. Even specificity within these categories occurs with, for example, some burrowing species tending to inhabit specific grain-size sediments [37, 39]. Such habitat specificity may restrict contaminant exposure vectors and influence trophic uptake as does the relatively small home ranges of nymphs [6, 31].

Despite being relatively closely related, we predicted that differential accumulation of trace elements among dragonfly genera would result from these differences in body form, biology, and ecological habits. In particular, differences in habitat use involving greater exposure to sediments, especially fine sediments, may cause divergence of trace element accumulation patterns. This prediction stems from contaminants in a lotic systems often being stored in stream sediments, particularly fine sediments of depositional zones. More specifically, our objectives were to (1) evaluate whether trace element accumulation differed among 8 syntopic lotic dragonfly genera with varying body forms and ecological habits from a CCW contaminated stream (2) further evaluate whether the level of genus is an appropriate taxonomic level for taxonomic and spatial comparisons (3) compare trophic position (*δ*^15^N) and carbon sources (*δ* ^13^C) among dragonfly nymphs and examine influences of body size (4) assess the relationships of trace element accumulation with body size, trophic position, and carbon sources.

## Methods

### Study sites

Study site descriptions are available in Fletcher et al. [23, 40, 41] and briefly summarized here. Our study stream, Beaver Dam Creek is located on the Savannah River Site (SRS), an 801 km^2^ National Environmental Research Park operated by the U.S. Department of Energy since 1951. Beaver Dam Creek is a sandy bottomed, blackwater tributary to the Savannah River that has been impacted by a coal fired power plant operated in its headwaters for over 55 years [42]. The primary structure in our study stream was wood debris and root masses; macrophytes were rare. Two sites on BDC were selected that differed in geomorphology, flood regime, and distance to primary CCW contaminant sources. Much of upstream Site A was channelized and all deeply incised with an inactive floodplain. Site A was also closer to the ash basin and coal pile runoff outfalls that represented primary contaminant sources. Downstream Site B was less deeply incised with an active floodplain that was flooded by both BDC and the Savannah River. Site B was also more distant to the outfalls.

Detailed description of sediment sampling and subsequent analyses and results are reported in [23]. Trace element analysis of the sediment data found that highest levels of most elements were found in depositional areas of the stream and the highest levels were found at Site B [23]. In contrast, trace element concentrations in runs, which spatially make up a larger area of sandhills streams such as BDC, generally decreased with distance from upstream sources [23]. These sediment data represent a single sampling effort and as such may not adequately portray the temporal exposure of the dragonflies that is integrated into their levels of accumulation. BDC receives excessive stormwater runoff from impervious surfaces that will pulse particularly fine sediments and organic matter through the system. Consequently we did not attempt to directly correlate these sediment values and concentrations obtained from the various dragonfly genera. However the results presented below do demonstrate site specific differences and indicate that previous exposure within a site does affect tissue accumulation patterns both within and between genera of dragonflies.

### Study organisms

Dragonflies are a diverse group distributed across every continent except Antarctica [37]. In North America alone, 9 families and 69 genera have been recorded [43]. Along with their ubiquitous geographic distributions, the aquatic nymphs inhabit diverse habitats ranging from water standing in tree holes, and temporary or permanent lentic waters to lotic systems from small headwater tributaries to large rivers [37]. Further, within a water body, dragonfly nymphs can exhibit a wide range of body forms and sub-habitat use. The influence of voracious predation by dragonfly nymphs can range from influencing prey feeding behavior and habitat use to prey survival which consequently can be influential on shaping aquatic community compositions [44, 45, 46, 47]. Nymphs are also an important prey item to a variety of aquatic, semi-terrestrial, or terrestrial predators [48, 49].

We collected and analyzed a total of 8 genera belonging to 4 families: Gomphidae (5 genera), Aeshnidae (1 genus), Corduliidae (1 genus), and Macromiidae (1 genus) (Table 1). Four of the 5 gomphid genera (*Dromogomphus*, *Gomphus*, *Progomphus*, and *Stylurus*) have relatively compact bodies well adapted for burrowing and live shallowly buried in stream sediments. However the long legged gomphid *Hagenius* with a palmate body form is a sprawler that typically hides among leaf litter and detritus. Similarly the macromiid genus *Macromia* exhibit a flat, palmate body form with long spider-like legs also suited for sprawling upon the stream bottom typically in slower stream habitats. Aeshnidae nymphs are long, slender bodied climbers which lurk in vegetation or wood debris while stalking prey. The climber *Boyeria* frequently clinging to small sticks or roots with which it is well camouflaged. *Epitheca* characterized as climber-sprawler [50] is intermediate in habits between the sprawling *Macromia* and climber *Boyeria*. It has a moderately broad body form and moderately long legs that may allow more versatile habitat use. *Epitheca* were frequently kicked from root masses in BDC. Five of these genera have Tolerance Values listed in the North Carolina Biotic Index (NCBI). Tolerance Values are indices ranging from 0–10 that indicate how sensitive taxa (Species or genera) are to degraded stream water quality [51]. Although these indices do not directly indicate sensitivities to trace element contamination, a low number indicates a more generally sensitive species to water quality impairments. *Macromia*, *Boyeria*, *Dromogomphus*, *Gomphus*, and *Hagenius* had Tolerance Values of 6.7, 6.3, 6.3, 6.2, and 4.0 respectively [51].

**Table 1:** Odonate genera, habitat use category, size classes, and numbers of composites analyzed for each site.

Odonate taxa	Habit	Size class	Min. size (mm)	Max. size (mm)	Site A *n*	Site B *n*	Total *n*
**Gomphidae**							
* Dromogomphus*	Burrower	1	12	17	1	1	2
		2	18	22	3	1	4
		3	23	27	3	4	7
		4	30	33	2	1	3
		5	38	40	2	1	3
* Stylurus*	Burrower	2	18	25	0	1	1
		3	28	33	2	2	4
		4	35	38	1	0	1
* Gomphus*	Burrower	1	12	15	1	3	4
		2	17	18	1	1	2
		3	21	24	2	1	3
* Progomphus*	Burrower	1	17	21	1	0	1
		2	24	28	3	0	3
* Hagenius*	Sprawler	1	20	21	1	1	2
		2	25	27	0	2	2
		3	42	42	1	0	1
**Macromiidae**							
* Macromia*	Sprawler	0	8	10	0	1	1
		1	11	14	2	4	6
		2	15	18	6	6	12
		3	21	21	0	1	1
		4	24	26	4	5	9
**Corduliidae**							
* Epitheca*	Climber-	1	8	10	0	1	1
	sprawler	2	11	14	2	4	6
		3	15	18	6	6	12
		4	21	21	0	1	1
**Aeshnidae**							
* Boyeria*	Climber	0	12	17	1	0	1
		1	18	23	3	1	4
		2	24	29	5	5	10
		3	30	34	2	5	7
**Totals**					52	51	103

### Tissue collection and handling

Odonate nymphs were collected by seine and dip net (3.2 mm mesh) between 14 January and 11 May 2011. All nymphs were held for 24 h in the laboratory to allow depuration [24, 52]. A simulated stream current was created by deflecting air bubbles from an air-stone in plastic tubs containing sieved BDC stream water. Taxa were sorted by size before being placed in the tubs to reduce predation. Nymphs were subsequently rinsed in Milli-Q water (18 MΩ deionized water) prior to freezing in sterile whirl-paks. We recognize that this washing technique may not remove all elements from the exoskeleton surfaces, but as each individual was treated similarly patterns, if they exist, would be due to taxonomic or site differences. Samples were later thawed, identified to genus, body length measured, lyophilized, and dry whole body mass attained. Length frequency analysis established size classes within each taxon and individuals were composited within size classes (Table 1) to acquire sufficient mass for trace element analyses. Some size classes did not yield sufficient mass for analyses. The average individual body mass was calculated for each composite. Composites comprised 1–38 individuals with larger numbers required to acquire sufficient mass of small size categories. Lyophilized composite samples were ground to a fine powder, and homogenized. Approximately 50 or 250 mg of dry sample was used for digestion depending upon size of digestion vessel used. We used 55 mL vessels for larger mass composites and 10 mL vessels for smaller composites. Previous analyses (A. H. Lindell and T. Murphy, unpublished data) indicated comparable results using the different vessel sizes with subsamples from a common sample. Trace metal–grade nitric acid (HNO_3_) was added (10 mL to the 55 mL vessels and 1 mL to the 10 mL vessels) to samples in pre-cleaned Teflon vessels before digestion in a microwave (MARS Xpress, CEM Corporation, Matthews, NC) with heating steps at 185°C over 15 min at 100% power, followed by 10 min at hold and a 5 min cool down cycle. After digestion with HNO_3_, samples were brought to a final volume of 7 mL (for 10 mL vessels)– 15 mL (for 55 mL vessels) with Milli-Q water.

### Trace element and stable isotope analyses

Trace element analysis of 16 elements (Be, V, Cr, Ni, Cu, Zn, As, Se, Sr, Cd, Sb, Cs, Ba, Hg, Tl, and Pb) was performed by inductively coupled plasma-mass spectroscopy (Nexion 300X ICP-MS; Perkin Elmer, Norwalk, CT, USA) on diluted samples (55 mL vessels = 0.6 mL digested sample with 9.2 mL Milli-Q water and 0.2 mL 2 μg/g Au in 3% UHP HNO_3_; 10 mL vessels = 1.5 mL digested sample with 3.4 mL Milli-Q water and 0.1 mL 2 μg/g Au in 3% UHP HNO_3_). A total of 103 odonate composites (Site A *n* = 52; Site B *n* = 51) were analyzed (S1 Table). We used external calibration standards (High-Purity Standards, Charleston, SC, USA) covering a range of 0.5–500 ppb for As, Ba, Be, Cd, Cs, Cr, Cu, Ni, Pb, Sb, Se, Sr, Tl, V, and Zn. Hg standards (Inorganic Ventures, Christiansburg, VA, USA) ranged 0.5–10 ppb. Certified reference material (LUTS-1 and TORT-2; National Research Council, Ottawa, ON, Canada) and blanks were included in digestion and analysis procedures for quality control. Mean percent recovery for elements in certified reference materials were: V (106%), Cr (68%), Ni (82%), Cu (105%), Zn (97%), As (91%), Se (101%), Sr (97%), Cd (104%), Hg (217%), and Pb (122%). Data were not corrected for percent recovery. Mean instrument detection limits (μg/g) were as follows: Be (0.356), V (0.357), Cr (0.376), Ni (0.431), Cu (0.297), Zn (2.20), As (0.788), Se (1.61), Sr (0.442), Cd (0.462), Sb (4.19), Cs (0.381), Ba (0.355), Hg (1.40), Ti (0.459), and Pb (0.401). Mercury recoveries were excessively high, but concentrations were not further evaluated because nearly all composites were still below detection limits. All element concentrations are presented in μg/g on a dry mass basis.

Stable isotope analyses performed on subsamples from each composite evaluated total %C and total %N content and C and N stable isotope signatures for the dragonfly tissue. A Finnigan Delta Plus mass spectrometer (Thermo-Finnigan, Bremen, Germany) in the Stable Isotope & Soil Biology Laboratory, Odum School of Ecology, University of Georgia was used. Isotope ratios were expressed in the delta (δ) format: δ^13^C or δ^15^N (units of ‰) = (R_sample_—R_standard_ /R_standard_) X 1000, where R is the ^13^C:^12^C ratio or ^15^N:^14^N ratio. A bovine standard was referenced against an international standard, and precision averaged ≤0.1%.

Because the isotopic signatures of aquatic primary producers can be difficult to ascertain, freshwater mussels have been used as integrators of primary production to convert the *δ*^15^N to a trophic position using the equation: (Trophic position = [(Predator *δ*^15^N –Herbivore *δ*^15^N)/3.4] + C) where "C" equals the position of the organisms used to calibrate trophic position [53, 54, 55, 56]. Such a conversion aids in standardizing the stable isotope signature among sites by accounting for differences in basal signatures. Mussels are rare in BDC, therefore in calculations of this equation, we used the site specific average of the scraper/collector *M*. *modestum* and deposit/filter feeding *C*. *fluminea*, to provide a trophic baseline as described previously [23, 41]. These calculations assume a ^15^N trophic fractionation of 3.4‰ for lack of site specific data. We acknowledge that fractionation rate can be taxa or habitat specific and enrichment rates may range from < 2 to 5‰. Furthermore the use of inappropriate fractionation ratio for specific trophic transfers may lead to misinterpretation of results [57, 58] and it is becoming increasingly apparent that fractionation rates are often lower than the 3.4‰ [58, 59, 60, 61, 62, 63, 64, 65, 66]. However with no site specific fractionation rates we use the 3.4‰ for our comparisons. Even though the accuracy of the absolute trophic position number is in question, standardizing the *δ*^15^N among sites for differences in basal signatures will improve spatial comparisons.

### Statistical analyses

Analysis of Variance (ANOVA) followed by Tukey pairwise comparisons compared δ^13^C and trophic position among habitat use categories and genera. Significant interaction terms in initial models including genus and site, prompted comparison of genera within sites separately. *Progomphus*, only collected from Site A, was excluded from the initial models containing both sites, but included in Site A models. The relationships between δ^13^C and trophic position with body mass were evaluated with Spearman Rank correlation coefficients. Element concentrations in composited whole body samples of 8 odonate genera were compared. Concentrations below detection limit (BDL) in at least 99% of the composites (Sb and Hg) did not require further analyses. Concentrations BDL also occurred in Be (42%), Cd (11%), Cs (50%), and Tl (64%). Thallium was excluded from parametric analyses. For statistical analyses, we replaced BDL concentrations with 50% of the mean detection limit. Distributions of element concentrations were improved by Log_e_ transformation prior to statistical analysis. Statistical comparisons were conducted with SYSTAT^®^ statistical package (version 13.00.05/2009, SYSTAT Software Inc., San Jose) and largely follow previously described methods [23, 40]. Correlation of a large number of element pairs revealed in a Spearman Rank correlation coefficient matrix prompted employment of Principal Components Analysis (PCA) to summarize the concentrations of 13 elements (Ni, Ba, Sr, V, Cu, Be, Cd, Cr, As, Se, Pb, Zn, and Cs). PCA reduced dimensionality of the data, and simplified comparison of element accumulation among genera and sites. The number of interpretable principal components was determined from component eigenvalues and scree plots. We employed a varimax rotation of the axes that tends to distribute the amount of variation explained more evenly across the set of components [67]. Factor scores for each component were saved for further analyses. Analysis of Variance (ANOVA) followed by Tukey pairwise comparisons (pwc) compared factors scores among genera. As with δ^13^C and trophic position analyses, initial models included genus, site and the associated interaction, but because the interaction was again usually significant for these analyses, sites were further analyzed separately. Again, *Progomphus* only collected from Site A, was only included in the Site A models. Relationship of trace element accumulation with body size, trophic position and δ^13^C was evaluated with linear regression.

## Results

### Stable isotope analyses

**Fig 1:**
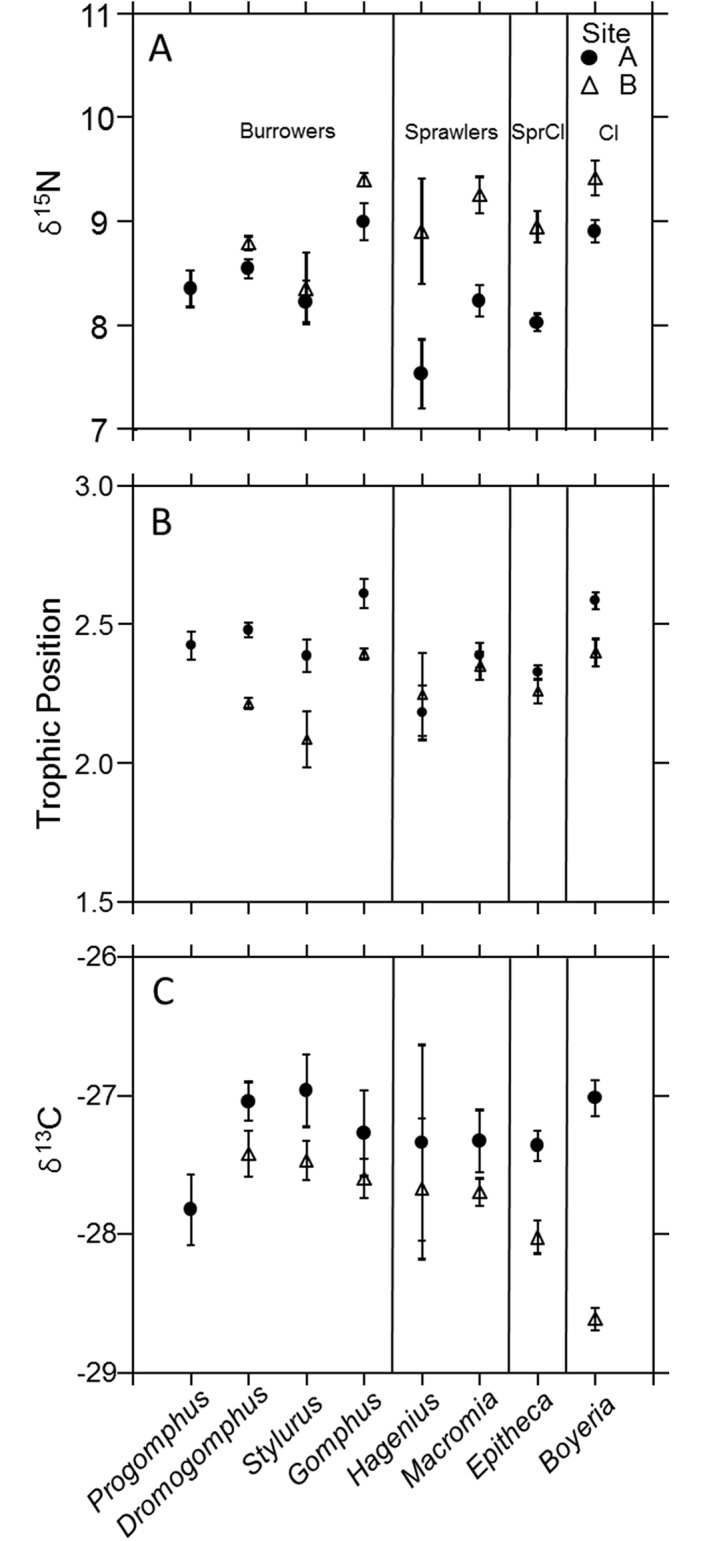
Trophic level and carbon sources. (A) Mean δ^15^N and (B) mean trophic position, and (C) mean δ^13^C compared among dragonfly nymph genera within each site. Error bars represent ± 1 SE.

For the genera collected at Site A (*Progomphus*, *Dromogomphus*, *Stylurus*, *Gomphus*, *Hagenius*, *Macromia*, *Epitheca*, and *Boyeria*), the *δ*^15^N spanned narrow ranges of 0.70, 1.08, 0.48, 0.63, 0.36, 1.34, 0.67, and 1.01‰ within genera, respectively. Within the genera collected in Site B (*Dromogomphus*, *Stylurus*, *Gomphus*, *Hagenius*, *Macromia*, *Epitheca*, and *Boyeria*), the *δ*^15^N spanned ranges of 0.48, 0.84, 0.36, 1.19, 1.64, 2.02, and 1.58‰. Across genera, averages of *δ*^15^N spanned ranges of 1.47‰ in Site A and 1.07‰ in Site B. Within these relatively narrow ranges, differences in *δ*^15^N among genera occurred, especially in Site A. Analysis of variance revealed *δ*^15^N differing among genera and between sites with a significant interaction (*R*^2^ = 0.63, genus *p* < 0.01, site *p* < 0.01, genus*site *p* < 0.01; Fig 1A). Generally, the taxa from the upstream site were more depleted of ^15^N, but different patterns among taxa were noted between sites. Despite Site A generally being more depleted with ^15^N, when standardized to basal signatures, trophic positions of *Dromogomphus*, *Stylurus*, *Gomphus* and *Boyeria* were actually higher in this site (*R*^2^ = 0.58, genus *p* < 0.01, site *p* < 0.01, genus*site *p* = 0.01; Fig 1B). In contrast, trophic position of *Hagenius*, *Macromia*, and *Epitheca* did not differ between sites. Mean trophic calculations ranged from a low of 2.18 in *Hagenius* to a high of 2.61 in *Gomphus* in Site A and a low of 2.08 in *Stylurus* to a high of 2.40 in *Boyeria* in Site B. This corresponds to the low level of variability in *δ*^15^N. Due to this spatial variability, trophic position was compared among genera within sites.

The trophic hierarchy in Site A ranged from the climber *Boyeria* and burrower *Gomphus* highest to the sprawler *Hagenius* and climber-sprawler *Epitheca* that tended to be lowest, with other genera overlapping and intermediate (*R*^2^ = 0.63, genus *p* < 0.01; Fig 1A and 1B). Pairwise comparisons indicated the gomphid genus *Hagenius* to trophically align more closely with the other sprawler and climber-sprawler than with burrowing members of its own family. Despite differences among genera (*R*^2^ = 0.34, genus *p* < 0.01), dragonfly nymph genera showed less trophic differentiation in Site B (Fig 1A and 1B). Again, the climber *Boyeria* and burrower *Gomphus* appeared highest, but with *Stylurus* and *Dromogomphus* lowest.

Based on Spearman Rank correlation coefficients, whether examining all composites together, combined by site, or by individual genera, no correlation between body size (dry mass) and trophic level was found (*p* > 0.05). Thus body size did not appear to influence trophic level.

For the genera collected at Site A *Progomphus*, *Dromogomphus*, *Stylurus*, *Gomphus*, *Hagenius*, *Macromia*, *Epitheca*, and *Boyeria*, the *δ* ^13^C variably spanned ranges of 0.96, 1.47, 0.61, 1.21, 0.77, 2.12, 0.91, and 1.36‰, respectively. The *δ* ^13^C of the 7 genera (*Progomphus*, *Dromogomphus*, *Stylurus*, *Gomphus*, *Hagenius*, *Macromia*, *Epitheca*, and *Boyeria*) collected in Site B, ranged 1.27, 0.37, 0.69, 1.32, 0.72, 1.32, and 0.98‰. Consequently, the *δ* ^13^C genus means ranged 0.86‰ in Site A and 1.19‰ in Site B. The *δ*^13^C differed among genera and between sites with a significant interaction (*R*^2^ = 0.63, genus *p* < 0.01, Site *p* < 0.01, Genus*Site *p* < 0.01; Fig 1C). The *δ*^13^C did not statistically differ among genera in Site A (*R*^2^ = 0.22, genus *p* = 0.11; Fig 1C), but did differ among some genera in Site B (*R*^2^ = 0.63, genus *p* < 0.01; Fig 1C) with the climber *Boyeria* more depleted of ^13^C than all other genera. From Fig 1C the climber-sprawler *Epitheca* appears more depleted of ^13^C than the other genera, but was only significantly more depleted than *Dromogomphus*. Interestingly, carbon sources utilized by the climber-sprawler *Epitheca* and climber *Boyeria* markedly diverged between sites as these groups differentiated more in Site B (Fig 1C).

The *δ*^13^C was not correlated to body size across all composites, but was negatively correlated when all genera from Site B were combined (p < 0.05). Additionally, within genera, the *δ*^13^C was positively correlated to body size in *Dromogomphus* (*p* < 0.01) and negatively correlated to body size in the climber *Boyeria* (*p* < 0.01). These correlations suggest changes in carbon source with increased body size, but different relationships in *Dromogomphus* and *Boyeria*.

### Trace element accumulation among sites and genera

Antimony and Hg did not substantially accumulate in dragonfly nymphs with at least 99% of composites BDL. Only a single *Hagenius* composite contained a detectable concentration of Hg. Thallium was also BDL in 64% of the composites with average concentrations less than 0.5 μg/g in all genera and a highest recorded concentration of 0.774 μg/g in a Site B *Epitheca* composite.

**Fig 2:**
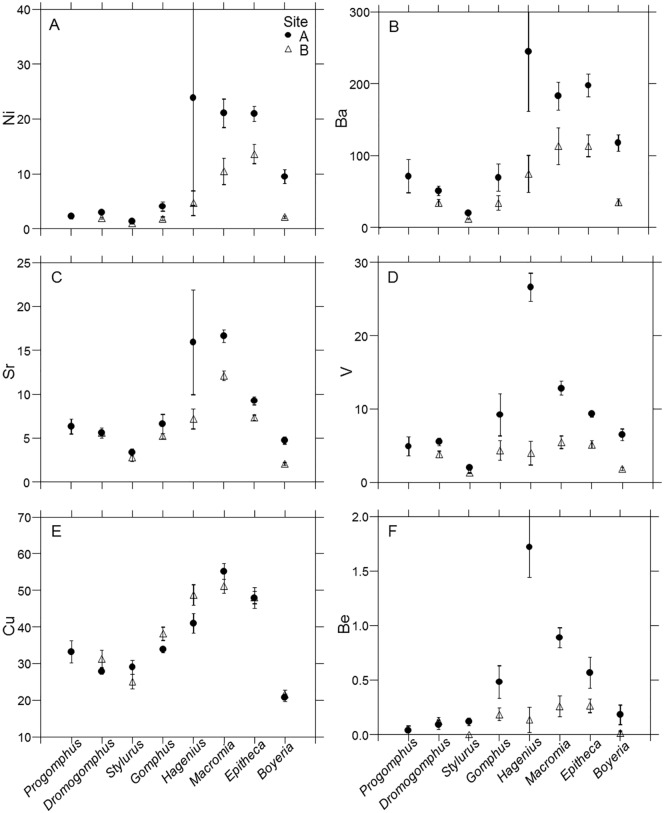
Trace element accumulation. (A-F) Mean whole body concentration of Ni, Ba, Sr, V, Cu, and Be (μg/g) for each dragonfly nymph genus within each site. Error bars represent ± 1 SE.

**Fig 3:**
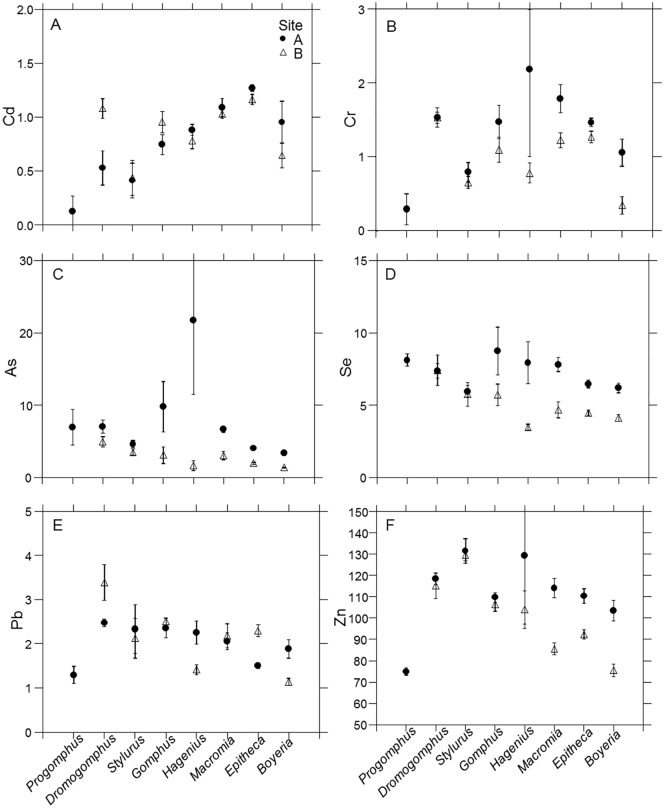
Trace element accumulation. (A-F) Mean whole body concentration of Cd, Cr, As, Se, Pb, and Zn (μg/g) for each dragonfly nymph genus within each site. Error bars represent ± 1 SE.

**Fig 4:**
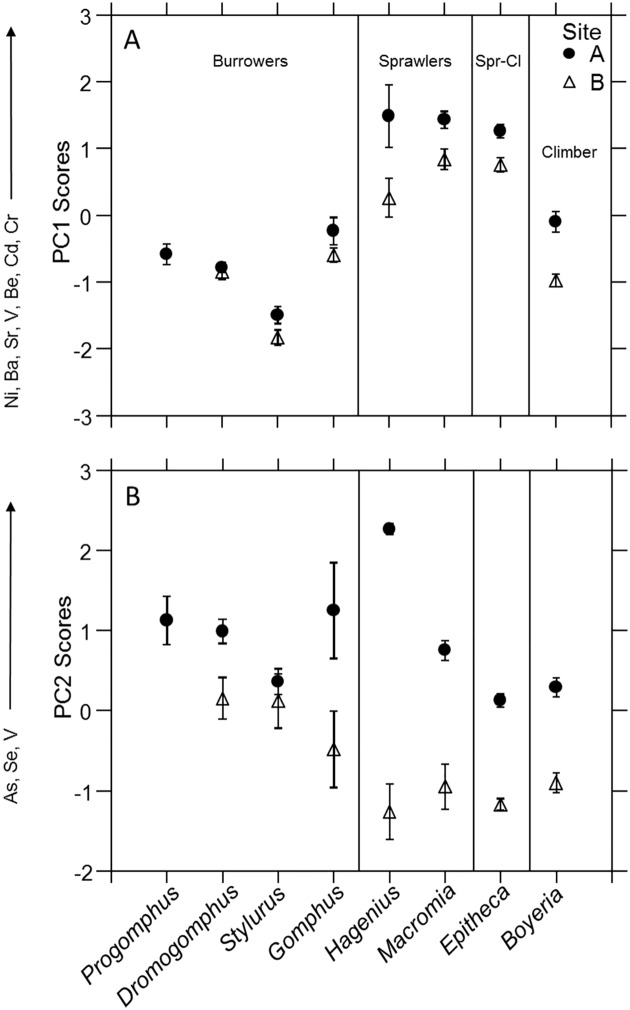
Multi-element accumulation patterns of PC1 and PC2 elements. (A) Mean PC1 and (B) mean PC2 factor scores from the whole body trace element concentration analysis for each dragonfly nymph genus within each site. Error bars represent ± 1 SE.

**Fig 5:**
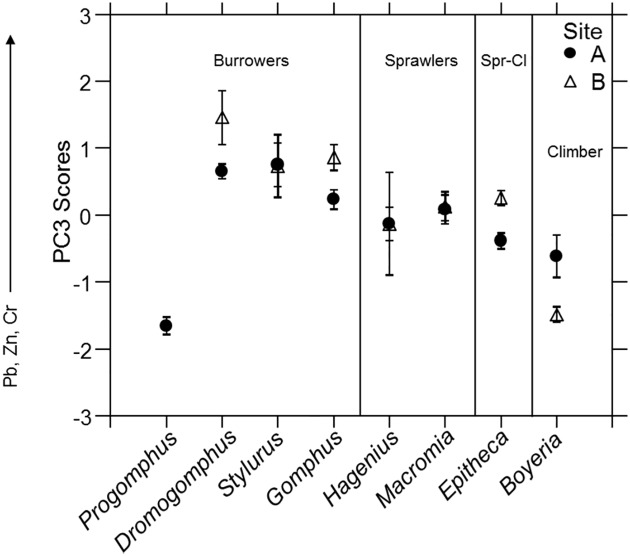
Multi-element accumulation patterns of PC3. Mean PC3 factor scores from the whole body trace element concentration analysis for each dragonfly nymph genus within each site. Error bars represent ± 1 SE.

The first 4 principal components accounted for 83% of the variation in the data (Table 2). Patterns in factor scores reflect the correlation of elements to each principal component. More specifically here, patterns in factor scores among genera and between sites generally reflect the patterns in accumulation of elements loading on the component (Figs 2, 3, 4 and 5). Generally, the stronger the loading on a component by an element, the more closely the pattern of accumulation follows that illustrated in the factor scores. Principal Component 1 (PC1) explained 36.4% of the variance with 8 elements (Ni, Ba, Sr, V, Cu, Be, Cd, and Cr) loading positively (Table 2). Accumulation of PC1 elements differed between sites and among genera, but with a significant interaction term (*R*^2^ = 0.90, genus *p* < 0.01, site *p* < 0.01, genus*site *p* < 0.01; Fig 4A). Specifically the three burrowing genera did not differ between sites (pwc *p* > 0.94). In contrast, the upstream Site A sprawlers *Hagenius* and *Macromia* (pwc *p* < 0.05), climber, *Boyeria* (pwc *p* < 0.01), and possibly the climber-sprawler *Epitheca* (pwc *p* = 0.08) all accumulated higher levels than the same taxa found in Site B.

**Table 2:** Loadings of body concentrations of trace elements on the first three principal components. For clarity, we include only loadings > 0.3 or < -0.3.

	PC1	PC2	PC3	PC4
Ni	0.943			
Ba	0.911			
Sr	0.847		0.300	
V	0.786	0.541		
Cu	0.755		0.333	
Be	0.676	0.351		0.468
Cd	0.573		0.333	
Cr	0.525	0.312	0.640	
As		0.905		
Se		0.882		
Pb			0.915	
Zn		0.471	0.689	
Cs				0.941
Eigenvalues	5.904	2.160	1.611	1.089
Percent total variance explained	36.443	19.523	16.662	10.163

Within Site A, accumulation of PC1 elements varied prominently among genera (*R*^2^ = 0.91, genus *p* < 0.01; Fig 4A). The two genera of sprawlers and the climber-sprawler accumulated the highest levels among all genera (pwc *p* < 0.05). In contrast, the burrower *Stylurus* accumulated the lowest concentrations (pwc *p* < 0.05). The other three burrowers *Progomphus*, *Dromogomphus*, and *Gomphus* along with the climber *Boyeria* generally accumulated intermediate concentrations. Despite some genera differing in accumulation between sites, accumulation of PC1 elements followed nearly an identical pattern among genera in both sites (*R*^2^ = 0.89, genus *p* < 0.01; Fig 4A; pwc *p* < 0.05).

The 2^nd^ principal component, PC2, explained 19.5% of the variance with positive loadings by As and Se (strong) and V (weak) (Table 2). PC2 factor scores differed among genera and between sites with a significant interaction (*R*^2^ = 0.76, genus *p* < 0.01, site *p* < 0.01, genus*site *p* < 0.01; Fig 4B). As with PC1 highest accumulations of these elements were found in taxa at Site A though not significantly in *Stylurus*. Greatest separation between sites was observed for *Hagenius*.

Patterns of accumulation of PC2 elements among genera notably differed between sites more than PC1 elements. For example, PC2 element concentrations in genera from Site A differed (*R*^2^ = 0.59, genus *p* < 0.01; Fig 4B), and differences occurred both within and among habitat use categories. *Hagenius* accumulated not only higher concentrations than the other sprawler and climber-sprawler, but higher than all genera. Some burrowers (*Progomphus*, *Dromogomphus*, and *Gomphus*) were also higher than *Epitheca* and *Boyeria* (pcw *p* ≤ 0.05). *Stylurus* again tended to accumulate lower concentrations than the other burrowers though not all comparisons were significant. Patterns among genera in Site B were very different with fewer significant differences between pairs of genera (*R*^2^ = 0.46, genus *p* < 0.01; Fig 4B). *Hagenius* did not accumulate the highest concentrations nor *Stylurus* the lowest in Site B.

Principal Component 3 (PC3) explains 16.7% of the variation with Pb loading the most strongly and weaker loadings of Zn and Cr (Table 2). PC3 scores significantly differed among genera and not sites, but the interaction was significant (*R*^2^ = 0.66, genus *p* < 0.01, site *p* = 0.23, genus*site *p* < 0.01). In Site A, PC3 scores differed among genera (*R*^2^ = 0.59, genus *p* < 0.01; Fig 5) with generally higher concentrations in burrowers. However, statistical differences in accumulation were found both among and within habitat use groups. The burrower *Progomphus* accumulated conspicuously lower concentrations than most other genera. Accumulation also differed among genera in Site B (*R*^2^ = 0.75, genus *p* < 0.01; Fig 5). Patterns of accumulation in genera were similar between sites. *Boyeria* had noticeably lower accumulation of all Site B genera (pcw *p* < 0.05).

The 4^th^ principal component (PC4) explained only 10.2% of the variance with Cs loading strongly on it (Table 2). PC4 scores did not differ among genera or between sites (*R*^2^ = 0.12, genus *p* = 0.52, site *p* = 0.12, genus*site *p* = 0.49). Consequently no further analysis was conducted on PC4 scores.

Spearman rank correlation coefficients revealed few consistent trends between the principal component scores and body mass, trophic position, or *δ*^13^C when all genera and sites are combined or when sites are analyzed separately. Two exceptions involved PC3 elements. PC3 elements negatively correlated with body mass with sites combined and individually in Sites A and B. PC3 elements also correlated positively with *δ*^13^C with sites combined and individually in Sites A and B. A positive correlation between *δ*^13^C and PC2 elements appeared to be driven by differences between sites as correlations were not significant in either Sites A or B when analyzed separately. Differences between sites influencing relationships are further illustrated by the positive correlation between PC2 elements and trophic position; this correlation was not significant in Site A, whereas a negative correlation occurred in Site B. Additional discrepancies between sites occurred with PC2 element concentrations only correlated in Site A (positively). Similarly PC1 element concentrations and trophic position was only correlated in Site A (negatively).

### Trace element intra-generic variation

**Fig 6:**
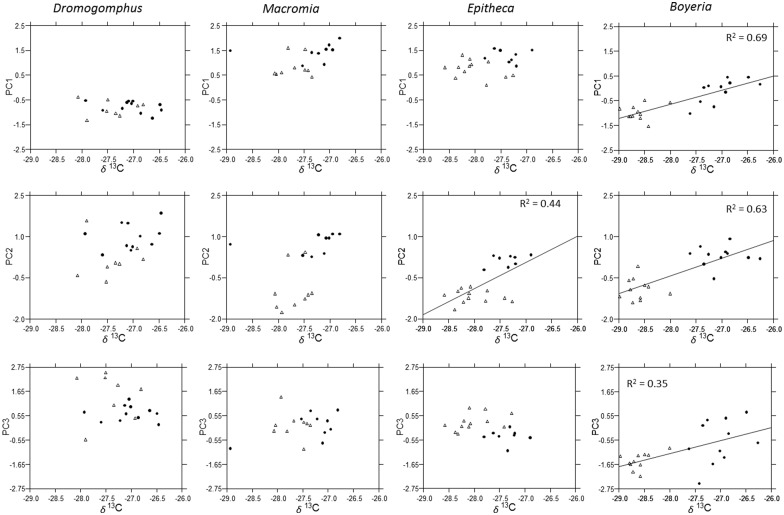
Multi-element relationships to *δ*^13^C. The relationship of factor scores from each principal component to *δ*^13^C for *Dromogomphus*, *Macromia*, *Epitheca*, and *Boyeria*. Regression lines and the associated R^2^ are illustrated for significant relationships (p < 0.05).

**Fig 7:**
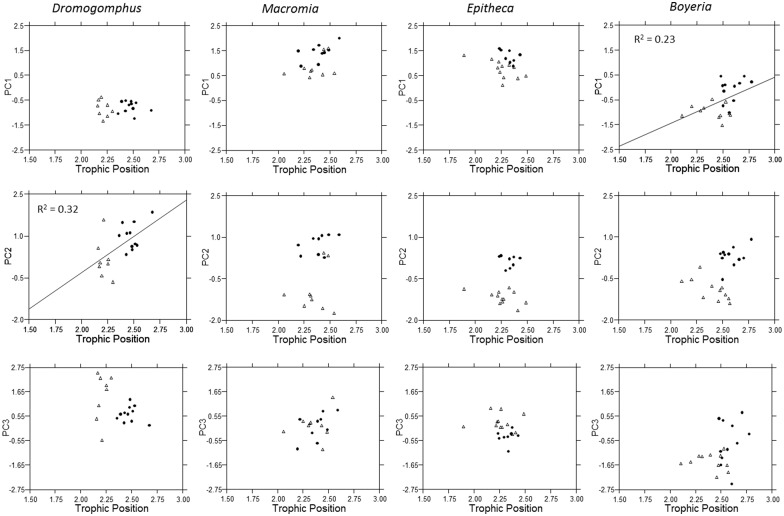
Multi-element relationships to trophic position. The relationship of factor scores from each principal component to trophic position for *Dromogomphus*, *Macromia*, *Epitheca*, and *Boyeria*. Regression lines and the associated R^2^ are illustrated for significant relationships (p < 0.05).

Accumulation of some trace element had a significant positive relationship to *δ*^13^C in two genera (PC1 *Boyeria*; PC2 *Epitheca*, *Boyeria*; Fig 6). However in all cases the patterns were driven by these genera differing in *δ*^13^C between sites. Individuals collected from upstream Site A accumulated higher concentrations of trace elements and were more enriched with *δ*^13^C. Trophic position was only significantly related to trace element accumulation in *Dromogomphus* and *Boyeria* (PC1 *Boyeria*; PC2 *Dromogomphus* and *Boyeria*; Fig 7) and was always positive. To varying degrees these relationships were influenced by trophic position differing between sites. For example, the positive relationship between accumulation of PC2 elements and trophic level in *Dromgomphus* and *Boyeria* is clearly driven by trophic level and accumulation being higher in Site A.

**Fig 8:**
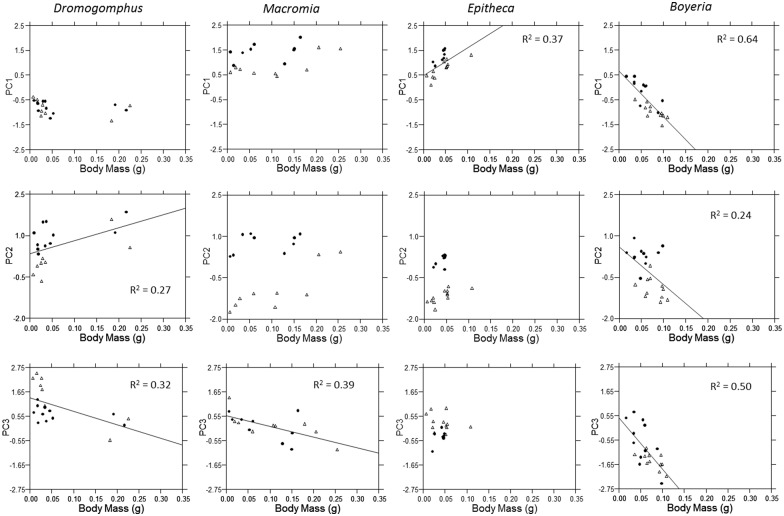
Multi-element relationships to body mass. The relationship of factor scores from each principal component to body mass for *Dromogomphus*, *Macromia*, *Epitheca*, and *Boyeria*. Regression lines the associated R^2^ are illustrated for significant relationships (p < 0.05).

Whether body size was positively or negatively related to trace element accumulation depended upon genus and element. A significant positive relationship was only apparent between accumulation and body size in *Epitheca* (PC1 elements; Fig 8) and *Dromogomphus* (PC2 elements). Trace element accumulation more commonly decreased with increasing body size (PC1 *Boyeria*; PC2 *Boyeria*; PC3 *Dromogomphus*, *Macromia*, and *Boyeria*; Fig 8). Unlike many of the relationships between accumulation and the stable isotope signatures, the relationships between body size and accumulation appear to be a more general response with the relationship often appearing relatively similar between sites (e.g. PC1 elements in *Boyeria*). In contrast however, the relationship between PC2 element accumulation and body size in *Boyeria* appears to be entirely driven by differences between sites. Clearly additional research is needed to explain these patterns.

## Discussion

### Inter-generic differences in trace element accumulation

Exposure to multiple element contaminations can result in a near community wide increase in trace element accumulation [1]. Indeed, exposure to the broad suite of trace elements associated with CCW in BDC resulted in higher accumulation of several elements (e.g. Ni, Ba, Sr, V, Be, Cr As, and Se) in all 8 dragonfly genera at the upstream site that was closer to contaminant sources. As has been shown for macroinvertebrates in general [1, 2, 3, 4] and more specifically with dragonfly nymphs in other systems [35, 33], genus and element specific patterns of accumulation were found among the 8 genera and 16 elements. No single genus consistently accumulated high or low concentrations of all elements, but as others have found [5, 6, 7], we were able to use multi-element trace element concentrations to distinguish patterns in accumulation among genera and sites.

Most consistent multi-element patterns were found in elements loading on PC1 (Ni, Ba, Sr, V, Cu, Be, Cd, and Cr) that represent a mix of essential and non-essential elements. Despite substantial variation in levels of accumulation among genera, these elements exhibited remarkably similar patterns of accumulation among genera when compared between sites. Moreover, genera within habitat use/body form groups also accumulated relatively similar concentrations compared to those with other forms and habits. In both sites, the two sprawler genera and sprawler-climber accumulated highest concentrations of PC1 elements. The four burrowing genera tended to accumulate lower concentrations with *Stylurus* accumulating lowest levels of all taxa. Differences in accumulation between genera of different habits can be substantial. For example, *Macromia* accumulated 10 times higher concentrations of Ni than *Stylurus* and 9 times higher Ba concentrations than *Boyeria* in Site A (Figs 2 and 3). The potential effects of habitat use and body form on accumulation may be illustrated by similar accumulation of the sprawlers and sprawler-climber even though the three genera belong to three different families. *Hagenius* sharing patterns with these taxa rather than the other gomphids represents an example of organismal biology influencing accumulation levels more than phylogeny.

Multiple factors have potential to produce the higher observed concentrations in the sprawlers. *Hagenius* shares both a broader, palmate body form and a similarity of habitat use with *Macromia* and *Epitheca*. The broader body form of these 3 genera, particularly *Macromia* and *Hagenius*, produces a greater surface area for their mass than do other genera of this study (Fletcher et al., unpublished data). It should be noted that with existing data, we could not differentiate the surface area to mass ratios of the other genera, but this should be the focus of future work. Even though other aspects of anatomy or physiology can have a strong influence on metal uptake [36, 68], greater surface area to mass ratio provides more interface for contact with contaminants and can increase accumulation, particularly in cases of elements that adsorb to the body surface [69, 70].

Additionally, a large portion of contaminants in a stream system are often stored in sediments [71, 72] that consequently can increase accumulation levels in associated aquatic macroinvertebrates [1, 35]. Moreover, habitat use involving greater exposure to bottom sediments can increase levels of accumulation [33, 35, 69]. Contaminants are heterogeneously distributed on stream bottoms with highest concentrations often occurring in depositional zones where finer sediments and organic matter settle out [23, 73, 74]. Consequently, the mesohabitat and associated sediment type occupied by an organism has potential to influence accumulation levels [23]. Although our sediment sampling [23] was not robust enough to correlate with observed patterns in these dragonflies it did demonstrate that there was incredible variation both within and between sites with higher concentrations in the depositional zones of BDC. More detailed study of the relationship physicochemical nature of sediments and biota accumulation in sandhills streams is warranted.

Sprawlers tend to inhabit slower depositional zones with more silty substrates [38], where higher levels of contaminants in the sediments have previously been found in BDC [23]. In addition to direct exposure to sediments, trophic uptake can be an important factor in contaminant accumulation [3, 75], consequently feeding on prey from these habitats may also increase accumulation. Consequently both body form and habitat use have the potential of explaining the higher levels of contaminants as do other biological differences such as efflux rates.

Arsenic, Se, and V represent common constituents of CCW [9, 16, 17, 18, 19, 20, 21, 22] and loaded (As, Se strongly, V weakly) on PC2. In 6 of 7 genera, these elements accumulated to higher concentrations in upstream Site A, closer to most contaminant sources. Despite consistently higher levels in Site A, patterns of accumulation among genera differed markedly between sites. Observed variability includes differences in accumulation both within and among habitat use categories. Unlike in PC1 elements, much variation of accumulation of PC2 elements occurred among the sprawler and sprawler-climber genera and whether concentrations were elevated depended upon genus and site. Arsenic tends to bind to the exoskeleton of aquatic insects [1, 33]. It was not only found that most arsenic was binding to the surface cuticle in *Gammarus pulex*, but that arsenic washed from the cuticle also had not undergone biological transformation [76]. However, arsenic concentrations in BDC dragonflies do not consistently follow expected accumulation patterns of being higher in genera with higher surface areas. Concentrations of these common constituents of CCW were more elevated in dragonfly larvae closer to contaminant sources, but the genus and site specific differentiation remains unexplained.

Accumulation of PC3 elements (Pb loaded strongly, Zn and Cr weakly) differed between sites in fewer genera. PC3 scores tended to by higher in burrowers, but over all there was generally less variability among genera. However the burrower *Progomphus* conspicuously accumulated lower concentrations. *Progomphus* occurs in particularly sandy habitats in coastal plain streams [38] that tend to have lower contaminant concentrations. Additionally Site B *Boyeria* accumulated lower concentrations of these elements in Site B. We observed both consistencies among and variation within habitat use/body form categories depending upon elements. Overall, these patterns illustrate the complex taxa and element specific variability inherent in trace element accumulation in aquatic insects and highlight the need of research explaining such patterns.

### Taxonomic resolution

The taxonomic level analyzed in studies of trace element accumulation in odonates varies extensively. Some studies analyzed organisms composited by order [77, 78] or suborder [5, 79, 80]. Others have composited by family, sometimes with comparisons between families [81], but others with only a single anisopteran family [7, 30, 82]. As in our results, differences in accumulation among genera have also been reported [21, 32, 33, 35, 83]. Two of these studies [32, 33] identified samples to species, but multiple species within a genus were not included. Overall, comparison of trace element accumulation among dragonfly species within a genus represents an understudied challenge.

In our analyses, collection of only one genus of aeshnid, macromiid, and corduliid prevented evaluation of trace element accumulation variation within these families. However, variation within a family is evident within the gomphids which includes not only differences between *Hagenius* and the burrowing genera, but differentiation among the burrowers. More over taxonomic variation can be greater than the spatial differences (Fig 4A). Based on the gomphids, clearly family or higher level taxonomic classifications of dragonflies are too high to make reasonable assessments of trace element accumulation. However in our data, variation within genera is generally considerably less than among genera (Figs 2, 3, 4 and 5). In a broader and more rigorous test of taxonomic resolution among aquatic insects, Cain et al. [1] concluded that genus would frequently be a suitable taxonomic level for contaminant accumulation studies. Similarly Buchwalter and Luoma [68] found that metal uptake rates can vary more within orders than between orders, but that species within a genus tended to be more similar. Though our data also suggests that genera are a reasonable choice of taxonomic level, future studies should compare species of dragonfly nymphs within a genus, but we fear that this may restrict work to later instars because structures commonly used in species level keys are only rudimentary in early instars.

### Trophic relationships

Trophic interactions within dragonfly communities have been reported in lentic systems, particularly those without predaceous fishes [44, 45]. Trophic hierarchies can stem from differences in trophic levels among species or from size classes within a species as cannibalism is well established among odonates [84, 85, 86]. Correspondingly, the *δ*^15^N has been shown to differ among wetland dwelling dragonfly genera [59]. Trophic relationships of dragonfly nymphs appear to be more poorly known among lotic dragonfly communities [87]. Within the observed relatively narrow *δ*^15^N ranges of this study, the climber *Boyeria* and burrower *Gomphus* tended to occupy the highest trophic level. Relationships among other genera varied spatially. At Site A, a clear trophic hierarchy was found with *Boyeria* and *Gomphus* at the top, the other burrowing genera intermediate, and sprawlers *Macromia* and *Hagenius* and climber sprawler *Epitheca* at the bottom. Interestingly fewer genera differed in Site B. Consistencies within a habitat use category included the gomphid *Hagenius* overlapping with the sprawler *Macromia* and climber sprawler *Epitheca*. However, the higher trophic level of *Gomphus* above the other burrowers demonstrates trophic disparity within habitat use categories.

Spatial variability of *δ* ^15^N included the general depletion of ^15^N in taxa from Site A. This pattern was observed in other BDC invertebrates [23], but not in more mobile large fishes [41]. Despite Site A generally being more depleted with ^15^N, when standardized to basal signatures, *Dromogomphus*, *Stylurus*, *Gomphus* and *Boyeria* actually occupied higher trophic positions in this site. Trophic position of sprawlers and sprawler-climber were similar between sites. No relationship was apparent between body size and trophic position for any genus which is consistent with results of Unrine et al. [21] for to wetland genera, *Tramea* and *Erythemis*.

Trophic calculations would suggest BDC dragonflies to represent less than 1 trophic position over *M*. *modestum* and *C*. *fluminea*, but caution is warranted because we have not confirmed the fractionation rate of ^15^N and a lower fractionation rate would increase the calculated trophic position. Four species of predatory fish were calculated to average trophic positions of 3.04, 3.50, 3.72, and 3.94 for Channel Catfish, Bowfin, Largemouth Bass, and Longnose Gar, respectively, from *δ*^15^N’s of 11.1, 12.7, 13.5, and 14.2 [41]. Whether comparing the calculated trophic position or the *δ*^15^N directly, the dragonflies fell between the predatory fish and herbivores as expected for a predatory aquatic insect.

Dragonfly nymph morphology, behavior, and gut contents have been used as evidence of exclusively predatory feeding and characterization as generalized opportunistic-carnivores [37, 43, 50, 88]. Non-animal material has generally been attributed to incidental ingestion or from the gut contents of ingested herbivores [37, 87]. Classification of dragonfly position based on stable isotope analysis (SIA) has been more varied. Some have classified dragonfly nymphs as predators [89, 90, 91], but others have considered nymphs likely feeding on both plant and animal material in some or all taxa [59, 79, 92]. In short, assimilation of plant material and the enrichment rate of ^15^N in trophic transfers between dragonfly nymphs and lower trophic levels do not appear to have been confirmed. Previous summaries have indicated that trophic fractionation can differ among trophic levels, even within the same food chain and even between taxa within the same trophic position [57, 58]. Differences in fractionation between various dragonflies and their prey appear to represent a critical data gap in understanding trophic ecology of lentic and lotic waters and should be the focus of future studies. Additionally, Unrine et al. [21] suggested possible explanations for lower than expected *δ*^15^N for dragonfly nymphs in the headwater wetlands in BDC to be the presence of multiple basal resources or the input of inorganic N from the Savannah River from which water is pumped through the BDC system.

Diets can differ among dragonfly species [37, 93] and these differences correspondingly result in dissimilar assimilation of C from different basal resources [59]. Similar to *M*. *modestum* and *C*. *flumineum* [23], dragonfly nymphs appeared to generally be more depleted of ^13^C in downstream Site B, again indicating consistent differences between these sites. In contrast to N, the *δ*^13^C did not differ among genera in Site A, but did in Site B. An interesting spatial pattern was revealed with the carbon sources of the climber-sprawler *Epitheca* and climber *Boyeria* diverging between sites as the *δ*^13^C became more depleted in Site B. Overall, the observed variability may indicate that these two genera may not be exposed to contaminants via the same trophic pathways and this could differ between sites. The *δ*^13^C also varied with nymph size within some genera, which suggests the change of basal resources as a nymph grows in size has the potential to differentiate trophic exposure of the result of the resource use shift.

### Intra-generic variation in trace element accumulation

Spatial variability within genera was evident in BDC with concentrations frequently lower at the downstream site further from primary contaminant sources. Similar spatial patterns were reported for *C*. *fluminea* in this system [23, 24]. However, the degree of variation depended not only upon the specific element in question, but also on odonate genus. PC1 and PC2 elements were generally lower at the downstream site, but burrowing genera generally exhibited lower spatial variability. In contrast, PC3 elements, particularly Pb, appeared higher in the downstream site in some genera. Similarly, concentrations of Pb appeared visually higher in *M*. *modestum* from the downstream site of this system [40]. Unrine et al. [21] reported trace element concentrations for 2 wetland libellulid genera (*Erythemis* and *Tramea*) from wetlands in the headwaters of BDC near the contaminant sources. Concentrations of Cd and Se were generally higher in these wetland odonates than in dragonflies from our downstream sites, but V concentrations were lower. Due to variability among genera in both studies, comparisons of Cu, Zn, As, and Pb were less consistent, again highlighting that differences between genera can be greater than differences between locations, depending upon element.

Influences of trophic position and carbon sources within dragonfly genera or even within genera of different aquatic insects appear to be an exceptionally understudied topic. Trophic position did not appear to have a strong influence on contaminant accumulation. Significant increases in accumulation was associated with higher trophic level in only *Boyeria* and *Dromogomphus* and then only for a subset of elements. The relatively narrow range of trophic position for most genera may be a factor influencing lack of patterns. In contrast, divergence of carbon sources (enrichment of ^13^C in Site A) in *Boyeria* was consistently associated with a change in accumulation in elements that loaded on all three principal components. In all cases, trace element accumulation increased as their diet became more enriched with ^13^C. Similarly the divergence of carbon sources in *Epitheca* was also associated with a significant increase in PC2 elements. Although not statistically significant the visual trend also seemed apparent in PC1 elements (Fig 6). Though we cannot confirm cause and effect, these consistent patterns illustrate a potential increase trace element accumulation resulting from a change in food chain basal resources.

Interestingly, body size in *Boyeria* had the opposite effect on accumulation as did trophic position. Smaller body size in upstream Site A was associated with higher accumulation of elements loading on all three principal components. Similar patters were observed for PC3 elements for *Dromogomphus* and *Macromia*. In contrast to the stable isotope data, body size often, but not always appeared to have a more general response that was not drive by spatial differences. Thus it seems the influence of body size on trace element accumulation is more likely to be generalized between sites, whereas spatial variability in basal carbon sources and trophic position may produce more local, site specific responses. Future work should explore these patterns. Overall, carbon sources, body size, and trophic position had the most distinctive influence on trace element accumulation in *Boyeria*. For this reason, *Boyeria* seems like an ideal candidate for future work to clarify these relationships and establish how well the observed patterns can be generalized to other locations. *Boyeria vinosa* which is a broadly distributed species, so much potential for comparative work exists.

Kormondy [94] also reported Zn concentrations inversely related to body size in a wetland dragonfly nymph species. Surface binding was suggested as the explanation because body surface area is proportionally less in larger individuals. However, Lavilla et al. [33] found more Zn to be incorporated inside the nymph rather than externally bound. Whether exposed in solution [94] or trophically can influence where a contaminant accumulates [3, 94]. The field collected samples analyzed by Lavilla et al. [33] would have likely been subjected to multiple exposure routes. Future work should further investigate these patterns in dragonflies. Overall, trends of trace element concentrations in our data set frequently increased with body size and appeared to be influenced by diet, but element and species specificity was again apparent.

## Conclusions

Within the narrow observed ranges of *δ*^15^N, trophic hierarchies were apparent within the dragonfly communities. Calculated trophic position fell between herbivorous invertebrates and large predatory fishes. Future work should establish ^15^N fractionation rates in dragonflies to allow more precise interpretation of SIA data. Size did not appear to influence trophic position. Trophic position rarely influenced trace element accumulation within genera and did not consistently correlate with accumulation among genera. Even though separated by relatively short distances, carbon sources utilized by the climber and sprawler-climber diverged between sites. An increase in trace element accumulation was associated with this divergence. Overall, body size and carbons sources were most consistently correlated to trace element accumulation in the climber *Boyeria*. Future work in other systems should investigate the potential of *Boyeria* being a model organism for such comparisons.

Higher trace element concentrations tended to accumulate in nymphs from the upstream site, closer to primary contaminant sources. However, differences in accumulation among genera within a site often exceeded differences between sites. Consequently, separating genera in comparisons of contaminant accumulation among different sites is critical to avoid inaccurate conclusions. The critical nature of separating dragonfly genera was also illustrated in Unrine et al. [21] that showed whether or not dragonfly nymphs accumulated higher concentrations of some elements than analyzed fishes, depended upon which of two nymph genera the fishes were compared to. Greater variation between genera than within genera in our data suggests genus as an acceptable unit of comparison in dragonfly nymphs.

We observed both consistencies in accumulation among as well as variation within habitat use categories. Although variation can occur even among closely related taxa, phylogenetically based patterns in trace element efflux or accumulation have been noted [1, 4]. Variability in accumulation often increases with taxonomic level resulting in greater variation within families than within genera [1]. Consequently greater similarity in accumulation among sprawlers of different families is particularly compelling. However, the need to refine habitat use categories and distinguish effects of body form and other physiological factors is apparent by variability within habitat use categories. For example, variability inherent in trace element accumulation among sediment types and variability in accumulation among burrowers warrants future work distinguishing potential differences in types of sediment inhabited by burrowers. Higher accumulation of several elements suggests sprawlers to frequently be the most sensitive indicator of bioavailable contaminants. However, sprawlers can often be relatively rare in lotic systems. Generally analyzing more genera of multiple habitat use groups will improve assessments of bioavailable contaminants in a streams system.

Our results underscore the element and taxa specific nature of trace element accumulation, but we provide evidence of accumulation of some trace elements differing among dragonflies that differ in body form and utilize different sub-habitats within a stream reach. However, whether taxa differed in accumulation depended upon element. Even within organisms exposed to the same trace element concentrations, differences in how a species uptakes, metabolizes, detoxifies, and effluxes specific elements can lead to vastly different levels of accumulation and different degrees of impact [2]. Moreover effects of body size and feeding habits can be species specific and all factors can be influenced by the hydrogeochemical nature of each element [1, 69]. Factors controlling the observed variation in trace element accumulation among and within genera of aquatic insects should continue to be the focus of future research.

## Supporting information

S1 Table Body mass (g), δ^15^N, δ^13^C, trophic position, and trace element concentrations (μ/g) for all analyzed dragonfly nymph composite samples for each genus from upstream Sites A and downstream Site B. (XLSX)

## References

[1] Cain DJ, Luoma SN, Carter JL, Fend SV. Aquatic insects as bioindicators of trace element contamination in cobble-bottom rivers and streams. 1992;Can J Fish Aquat Sci. 49: 2141–2154.

[2] Rainbow PS. Trace metal concentrations in aquatic invertebrates: why and so what? Environ Pollut. 2002;120: 497–507. 12442773 10.1016/s0269-7491(02)00238-5

[3] Luoma SN, Rainbow PS. Why is metal bioaccumulation so variable? Biodynamics as a unifying concept. Environ Sci and Tech. 2005;39: 1921–1931. 10.1021/es048947e 15871220

[4] Poteat MD, Garland T Jr, Fisher NS, Wang W, Buchwalter DB. Evolutionary patterns in trace metal (Cd and Zn) efflux capacity in aquatic organisms. Environ Sci Tech. 2013;47: 7989–7995. 10.1021/es401368u 23772993

[5] Corbi JJ, Trivinho-Strixino S, dos Santos A. Environmental evaluation of metals in sediments and dragonflies due to sugar cane cultivation in neotropical streams. Water Air Soil Poll. 2008;195: 325–333.

[6] Corbi JJ, dos Santos FA, Zerlin R, dos Santos A, Froehlich CG, Trivinho-Strixino S. Assessment of chromium contamination in the Monte Alegre stream: A case study. Braz Arch Biol Techn. 2011a;54: 613–620.

[7] Karimi R, Folt CL. Beyond macronutrients: element variability and multi-element stoichiometry in freshwater invertebrates. Ecology Letters. 2006;9: 1273–1283. 10.1111/j.1461-0248.2006.00979.x 17118001

[8] Ruhl L, Vengosh A, Dwyer GS, Hsu-Kim H, Schwartz G, Romanski A, et al The impact of coal combustion residue effluent on water resources: A North Carolina example. Environ Sci Tech. 2012;46: 12226–12233. 10.1021/es303263x 23020686

[9] Rowe CL, Hopkins WA, and Congdon JD. Ecotoxicological implications of aquatic disposal of coal combustion residues in the United States: a review. Environ Monit Assess. 2002;80: 207–276. 12503897 10.1023/a:1021127120575

[10] Rowe CL. Bioaccumulation and effects of metals and trace elements from aquatic disposal of coal combustion residues: Recent advances and recommendations for further study. Sci Total Environ. 2014;485–486: 490–496. 10.1016/j.scitotenv.2014.03.119 24742559

[11] Demirak A, Yilmaz F, Levent Tuna A, Ozdemir N. Heavy metals in water, sediment and tissues of *Leuciscus cephalus* from a stream in *southwestern Turkey*. Chemosphere. 2006;63:1451–1458. 10.1016/j.chemosphere.2005.09.033 16325225

[12] Donahue WF, Allen EW, Schindler DW. Impacts of coal-fired power plants on trace metals and polycyclic aromatic hydrocarbons (PAHs) in lake sediments in central Alberta, Canada. J Paleolimnol. 2006;35:111–128.

[13] Rath P, Panda UC, Bhatta D, Sahu KC. Use of sequential leaching, mineralogy, morphology and multivariate statistical technique for quantifying metal pollution in highly polluted aquatic sediments—A case study: Brahmani and Nandira Rivers, India. J Hazard Mater. 2009;163:632–644. 10.1016/j.jhazmat.2008.07.048 18762380

[14] Rowe CL, Hopkins WA, Coffman VR, Failed Recruitment of Southern Toads (*Bufo terrestris*) in a Trace Element-Contaminated Breeding Habitat: Direct and Indirect Effects That May Lead to a Local Population Sink. Arch Environ Contam Toxicol. 2001;40:399–405. 10.1007/s002440010189 11443372

[15] Luther L. Managing coal combustion waste (CCW): Issues with disposal and use. Congressional Research Service, CRS report for Congress 7–5700, R40544; 2010. pp. 1–26.

[16] Lemly AD, Skorupa JP. Wildlife and the Coal Waste Policy Debate: Proposed Rules for Coal Waste Disposal Ignore Lessons from 45 Years of Wildlife Poisoning. Environ Sci Tech. 2012;46:8595–8600. 10.1021/es301467q 22839645

[17] Cherry DS, Currie RJ, Soucek DJ. Ecotoxicological impact of coal combustion by-products (CCBs) In South Carolina/Virginia streams during the 1970s to early 1980s. In: Vories KC, Harrington A (eds.), State regulation of coal combustion by-product placement at mine sites: a technical interactive forum. Proceedings Published by the U.S. Department of the Interior, Office of Surface Mining and the Coal Research Center, Southern Illinois University, Carbondale, Illinois; 2004. pp. 193–212.

[18] Evans DW, Giesy Jr JP. Trace metal concentrations in a stream-swamp system receiving coal ash effluent. In: Wali MK (editor). Ecology and Coal Resource Development, Vol. 2. Proceedings of the International Congress on Energy and the Ecosystem, Grand Fork, ND. Pergamon Press, New York; 1978. pp. 782–790.

[19] Hopkins WA, Roe JH, Snodgrass JW, Jackson BP, Kling DE, Rowe CL, et al Nondestructive indices of trace element exposure in squamate reptiles. Environ Pollut. 2001;115: 1–7. 11586765 10.1016/s0269-7491(01)00098-7

[20] Snodgrass JW, Hopkins WA, Roe JH. Relationships among developmental stage, metamorphic timing, and concentrations of elements in bullfrogs (*Rana catesbeiana*). Environ Toxicol Chem. 2003;22: 1597–1604. 12836987

[21] Unrine JM, Hopkins WA, Romanek CS, Jackson BP. Bioaccumulation of trace elements in omnivorous amphibian larvae: implications for amphibian health and contaminant transport. Environ Pollut. 2007;149: 182–192. 10.1016/j.envpol.2007.01.039 17399874

[22] Metts BS, Buhlmann KA, Tuberville TD, Scott DE, Hopkins WA. Maternal transfer of contaminants and reduced reproductive success of southern toads (Bufo [*Anaxyrus*] *terrestris*) exposed to coal combustion waste. Environ Sci Tech. 2013;47: 2846–2853. 10.1021/es303989u 23406432

[23] Fletcher DE, Lindell AH, Stillings GK, Mills GL, Blas SA, McArthur JV. Spatial and taxonomic differences in trace element bioaccumulation in two herbivores from a coal combustion waste contaminated stream. Ecotox Environ Safe. 2014a;101: 196–204. 10.1016/j.ecoenv.2013.12.024 24507146

[24] Peltier GL, Wright MS, Hopkins WA, Meyer JL. Accumulation of trace elements and growth responses in *Corbicula fluminea* downstream of a coal-fired power plant. Ecotox Environ Safe. 2009;72: 1384–1391. 10.1016/j.ecoenv.2009.01.011 19272648

[25] Guthrie RK, Cherry DS. Pollutant removal from coal-ash basin effluent. Water Resour Bull. 1976;12: 889–902.

[26] Guthrie R K, Cherry DS. Trophic level accumulation of heavy metals in a coal ash basin drainage system. Water Resour Bull. 1979;15: 244–248.

[27] Cherry DS, Guthrie RK, Larrick SR, and Sherberger F. The influence of coal ash and thermal discharges upon the distribution and bioaccumulation of aquatic invertebrates. Hydrobiologia. 1979;62: 257–267.

[28] Córdoba-Aguilar A (ed). Dragonflies and Damselflies Model organisms for ecological and evolutionary research. Oxford, UK: Oxford University Press; 2008.

[29] Nummelin M, Lodenius M, Tulisalo E, Hirvonen H, Alanko T. Predatory insects as bioindicators of heavy metal pollution. Environ Pollut. 2007;145: 339–347. 10.1016/j.envpol.2006.03.002 16678317

[30] Corbi JJ, Froehlich CG, Trivinho-Strixino S, dos Santos A. Evaluating the use of predatory insects as bioindicators of metals contamination due to sugarcane cultivation in neotropical streams. Environ Monit Assess. 2011b;177: 545–554. 20711857 10.1007/s10661-010-1655-5

[31] Wiener J, Haro R, Rolfhus K, Sandheinrich M, and Route B. Protocol for monitoring and assessing methylmercury and organic contaminants in aquatic food webs. National Park Service, Great Lakes Inventory and Monitoring Network Technical Report No. GLKN/2008/version 1.0 (April 18, 2008).

[32] Tollett VD, Benvenutti EL, Deer LA, Rice TM. Differential toxicity to Cd, Pb, and Cu in dragonfly larvae (Insecta: Odonata). Arch Environ Con Tox. 2009;56: 77–84. 10.1007/s00244-008-9170-1 18421495

[33] Lavilla I, Rodríguez-Liñares G, Garrido J, Bendicho C. A biogeochemical approach to understanding the accumulation patterns of trace elements in three species of dragonfly larvae: evaluation as biomonitors. J Environ Monitor. 2010;12: 724–730. 10.1039/b920379f 20445862

[34] Worthen WB. The structure of larval odonate assemblages in the Enoree River Basin of South Carolina. Southeast Nat. 2002;1: 205–216.

[35] Corbi JJ, Froehlich CG, Trivinho-Strixino S, dos Santos A. Bioaccumulation of metals in aquatic insects of streams located in areas with sugar cane cultivation. *Quím Nova*. 2010;33: 644–648.

[36] Buchwalter DB, Cain DJ, Martin CA, Xie L, Luoma SN, and Garland T Jr. Aquatic insect ecophysiological traits reveal phylogenetically based differences in dissolved cadmium susceptibility. P Natl Acad Sci. USA 2008;105: 8321–8326. 10.1073/pnas.0801686105 PMC2448835 18559853

[37] Corbet PS. Dragonflies: Behavior and Ecology of Odonata. Ithaca, NY: Cornell University Press; 1999.

[38] Burcher C, Smock L. Habitat distribution, dietary composition and life history characteristics of odonate nymphs in a blackwater coastal plain stream. Am Midl Nat. 2002;148: 75–89.

[39] Worthen WB, Gregory S, Felten J, Hutton MJ. Larval habitat associations of *Progomphus obscurus* at two spatial scales (Odonata: Gomphidae). Int J Odonatol. 2004;7: 97–109.

[40] Fletcher DE, Lindell AH, Stillings GK, Mills GL, Blas SA, McArthur JV. Variation in trace-element accumulation in predatory fishes from a stream contaminated by coal combustion waste. Arch of Environ Con Tox. 2014b;66: 341–360. 10.1007/s00244-013-9984-3 24384693

[41] Fletcher DE, Lindell AH, Stillings GK, Mills GL, Blas SA, McArthur JV. Trophic variation in coastal plain stream predatory fishes. Southeast Nat. 2015;14: 373–396.

[42] Halverson NV, Wike LD, Patterson KK, Bowers JA, Bryan AL, Chen KF, et al. SRS environmental information document—SRS ecology chapter 5—streams, reservoirs, and the Savannah River. Section 5.2—Beaver Dam Creek drainage description and surface hydrology. WSRC-TR-97-0223; 1997. pp. 67–119.

[43] Needham JG, Westfall MJ Jr, May ML. Dragonflies of North America The odonata (Anisoptera) fauna of Canada, the continental United States, Northern Mexico and the Greater Antilles. 3rd ed Gainesville, Florida: Scientific Publishers; 2014.

[44] Benke AC. Interactions among coexisting predators—a field experiment with dragonfly larvae. J of Anim Ecol. 1978;47: 335–350.

[45] Morin PJ. Experiments with colonization history and fish predation. Ecology. 1984;65: 1866–1873.

[46] Thorp J, Cothran ML. Regulation of freshwater community structure at multiple intensities of dragonfly predation. Ecology. 1984;65: 1546–1555.

[47] Crumrine PW, Switzer PV, Crowley PH. Structure and dynamics of odonate communities: accessing habitat, responding to risk, and enabling reproduction In: Córdoba-Aguilar A(ed) Dragonflies and Damselflies. Model organisms for ecological and evolutionary research. Oxford, UK: Oxford University Press; 2008 pp 21–38.

[48] Kennedy CJ. The relation of American dragonfly-eating birds to their prey. Ecol Monogr. 1950;20: 103–142.

[49] Knight TM, McCoy MW, Chase JM, McCoy KA, Holt RD. Trophic cascades across ecosystems. Nature. 2005;437: 880–884. 10.1038/nature03962 16208370

[50] Merritt RW, Cummins KW, Berg MB (eds). An Introduction to the Aquatic Insects of North America, 4th ed. Dubuque, Iowa: Kendall/ Hunt Publishing Company; 2008.

[51] Lenat DR. A biotic index for the southeastern United States: derivation and list of tolerance values, with criteria for assigning water-quality ratings. J N Am Benthol Soc. 1993;12: 279–290.

[52] Wilson AL, Ryder DS, Watts RJ Stevens MM. Stable isotope analysis of aquatic invertebrate communities in irrigated rice fields cultivated under different management regimes. Aquat Ecol. 2005;39: 189–200.

[53] Cabana G, Rasmussen JB. Comparison of aquatic food chains using nitrogen isotopes. P Natl Acad Sci. 1996;93: 10844–10847. 10.1073/pnas.93.20.10844 PMC38243 8855268

[54] Vander Zanden MJ, Cabana G, Rasmussen JB. Comparing trophic position of freshwater fish calculated using stable nitrogen isotope ratios (^15^N) and literature dietary data. Can J Fish Aquat Sci. 1997;54: 1142–1158.

[55] Post DM, Pace ML, Hairston NG Jr. Ecosystem size determines food-chain length in lakes. Nature. 2000;405: 1047–1049. 10.1038/35016565 10890443

[56] Overman NC Parrish DL. Stable isotope composition of Walleye:^15^N accumulation with age and area-specific differences in *δ* ^13^C. Can J Fish Aquat Sci. 2001;58: 1253–1260.

[57] Jardine TD, Curry RA, Heard KS, Cunjak RA. High fidelity: isotopic relationship between stream invertebrates and their gut contents. J N Am Benthol Soc. 2005;24: 290–299.

[58] Caut S, Angulo E, Courchamp F. Variation in discrimination factors (Δ^15^N and Δ^13^C): the effect of diet isotopic values and applications for diet reconstruction. J Appl Ecol. 2009;46: 443–453.

[59] Molina CI, Gibon FG, Oberdorff T, Dominguez E, Pinto J, Marín R, et al Macroinvertebrate food web structure in a floodplain lake of the Bolivian Amazon. Hydrobiologia. 2011;663: 135–153.

[60] Gu B, Schelske CL, Hoyer MV. Stable isotopes of carbon and nitrogen as indicators of diet and trophic structure of the fish community in a shallow hypereutrophic lake. J Fish Biol. 1996;49: 1233–1243.

[61] Jepsen DB, Winemiller KO. Structure of tropical river food webs revealed by stable isotope ratios. Oikos. 2002;96: 46–55.

[62] Post DM. Using stable isotopes to estimate trophic position: models, methods, and assumptions. Ecology. 2002;83: 703–718.

[63] McCutchan JH Jr, Lewis WM Jr, Kendall C, McGrath CC. Variation in trophic shift for stable isotope ratios of carbon, nitrogen, and sulfur. Oikos. 2003;102: 378–390.

[64] Herwig BR, Soluk DA, Dettmers JM, Wahl DH. Trophic structure and energy flow in backwater lakes of two large floodplain rivers assessed using stable isotopes. Can J Fish Aquat Sci. 2004;61: 12–22.

[65] Kilham SS, Hunte-Brown M, Verburg P, Pringle CM, Whiles MR, Lips KR, et al Challenges for interpreting stable isotope fractionation of carbon and nitrogen in tropical ecosystems. Verhandlungen Internationale Vereinigung für Theoretische und Angewandte Limnologie. 2009;30: 749–753.

[66] Cremona F, Timm H, Agasild H, Tõnno I, Feldman T, Jones RI, et al Benthic foodweb structure in a large shallow lake studied by stable isotope analysis. Freshw Sci. 2014;33: 885–894.

[67] Wilkinson L, Blank G, Gruber C. Desktop data analysis with SYSTAT. Upper Saddle River, NJ: Prentice Hall; 1996.

[68] Buchwalter DB, Luoma SN. Differences in dissolved cadmium and zinc uptake among stream insects: mechanistic explanations. Environ Sci Technol. 2005;39: 498–504. 15707049 10.1021/es0404421

[69] Smock LA. The influence of feeding habits on whole-body metal concentrations in aquatic insects. Freshwater Biol. 1983;13: 301–311.

[70] Farag AM, Woodward DF, Goldstein JN, Brumbaugh W, Meyers JS. Concentrations of metals associated with mining waste in sediments, biofilm, benthic macroinvertebrates, and fish from the Coeur d’Alene River Basin, Idaho. Arch Environ Contam Toxicol. 1998;34: 119–127. 9469853 10.1007/s002449900295

[71] Cherry DS, Guthrie RK. Mode of elemental dissipation from ash basin effluent. Water Air Soil Poll. 1978;9: 403–412.

[72] Taylor KG, Owens PN. Sediments in urban river basins: a review of sediment–contaminant dynamics in an environmental system conditioned by human activities. J Soil Sediments. 2009; 9: 281–303.

[73] Shelton LR, and PD Capel. Guidelines for collecting and processing samples of stream bed sediment for analysis of trace elements and organic contaminants for the national water-quality assessment program. Surface Water Ambient Monitoring Program (SWAMP) Quality Assurance Management Plan, Appendix D: Open-file Report 94–458. U.S. Geological Survey, Denver, Colorado; 1994.

[74] Paul MJ, Meyer JL. Streams in the urban landscape. Annu Rev Ecol Syst. 2001;32: 333–365.

[75] Roy I, Hare L. Relative importance of water and food as cadmium sources to the predatory insect *Sialis velata* (Megaloptera). Can J Fish Aquat Sci. 1999;56: 1143–1149.

[76] Schaller J, Koch I, Caumette G, Nearing M, Reimer KJ, Planer-Friedrich B. Strategies of *Gammarus pulex* L. to cope with arsenic—Results from speciation analyses by IC–ICP-MS and XAS micro-mapping. Sci Total Environ. 2015;530–531:430–433. 10.1016/j.scitotenv.2015.06.015 26068228

[77] Karouna-Renier NK, Sparling DW. Relationships between ambient geochemistry, watershed land-use and trace metal concentrations in aquatic invertebrates living in stormwater treatment ponds. Environ Pollut. 2001;112: 183–192. 11234534 10.1016/s0269-7491(00)00119-6

[78] Casey RE, Simon JA, Atueyi S, Snodgrass JW, Karouna-Renier N, Sparling DW. Temporal trends of trace metals in sediment and invertebrates from stormwater management ponds. Water Air Soil Pollut. 2006;178: 69–77.

[79] Edwards PG, Gaines KF, Bryan AL Jr, Novak JM, Blas SA. Are U, Ni, and Hg an Environmental Risk within a RCRA/CERCLA Unit on the U.S. Department of Energy's Savannah River Site? Hum Ecol Risk Assess. 2014;20: 1565–1589.

[80] Arribére MA, Cambell LM, Rizzo AP, Arcagni M, Revenga J, Ribeiro Guevara S. Trace elements in plankton, benthic organisms, and forage fish of Lake Moreno, Northern Patagonia, Argentina. Water Air Soil Pollut. 2010;212: 167–182.

[81] Chumchal MM, Rainwater TR, Osborn SC, Roberts AP, Abel MT, Cobb GP, et al Mercury speciation and biomagnification in the food web of Caddo Lake, Texas and Louisiana, USA a subtropical freshwater ecosystem. Environ Toxicol Chem. 2011;30: 1153–1162. 10.1002/etc.477 21305578

[82] Scheuhammer AM, McNicol DK, Mallory ML, Kerekes JJ. Relationships between lake chemistry and calcium and trace metal concentrations of aquatic invertebrates eaten by breeding insectivorous waterfowl. Environ Pollut. 1997;96: 235–247. 15093423 10.1016/s0269-7491(97)00032-8

[83] Henderson BL, Chumchal MM, Drenner RW, Deng Y, Diaz P, Nowlin WH. Effects of fish on mercury contamination of macroinvertebrate communities of grassland ponds. Environ Toxicol Chem. 2012;31: 870–876. 10.1002/etc.1760 22278821

[84] Van Buskirk J. Competition, cannibalism, and size class dominance in a dragonfly. Oikos. 1992;65: 455–464.

[85] Wissinger S, McGrady J. Intraguild predation and competition between larval dragonflies: direct and indirect effects on shared prey. Ecology. 1993;74: 207–218.

[86] Crumrine PW, Crowley PH. Partitioning components of risk reduction in a dragonfly-fish intraguild predation system. Ecology. 2003;84: 1588–1597.

[87] Bo T, Fenoglio S, López-Rodríguez MJ, Tierno de Figueroa JM. Trophic behaviour of the dragonfly *Cordulegaster boltoni* (Insecta: Odonata) in small creeks in NW Italy. Entomologica Fennica. 2011;22: 255–261.

[88] Wallace JB, Cuffney TF, Lay CC, and Vogel D. The influence of an ecosystem-level manipulation on prey consumption by a lotic dragonfly. Can J Zoolog. 1987;65: 35–40.

[89] Kupfer A, Langel R, Scheu S, Himstedt W, Maraun M. Trophic ecology of a tropical aquatic and terrestrial food web: insights from stable isotopes (^15^N). J Trop Ecol. 2006;22: 469–476.

[90] Bennett PM, Hobson KA. Trophic structure of a boreal forest arthropod community revealed by stable isotope (d13C, d15N) analyses. Entomol Sci. 2009;12: 17–24.

[91] Clayden MG, Kidd KA, Wyn B, Kirk JL, Muir DCG, and O’Driscoll NJ. Mercury biomagnification through food webs is affected by physical and chemical characteristics of lakes. Envir Sci Tech. 2013;47: 12047–12053. 10.1021/es4022975 24099312

[92] Hicks BJ. Food webs in forest and pasture streams in the Waikato region, New Zealand: a study based on analyses of stable isotopes of carbon and nitrogen, and fish gut contents. New Zeal J Mar Fresh. 1997;31: 651–664.

[93] Blois C. The larval diet of three Anisopteran (Odonata) species. Freshwater Biol. 1985;15: 505–514.

[94] Kormondy EJ. Uptake and loss of zinc-65 in the dragonfly *Plathemis lydia*. Limnol Oceanogr. 1965;10: 427–433.

